# A healthy lifestyle is positively associated with mental health and well-being and core markers in ageing

**DOI:** 10.1186/s12916-022-02524-9

**Published:** 2022-09-29

**Authors:** Pauline Hautekiet, Nelly D. Saenen, Dries S. Martens, Margot Debay, Johan Van der Heyden, Tim S. Nawrot, Eva M. De Clercq

**Affiliations:** 1 Sciensano, Risk and Health Impact Assessment, Juliette Wytsmanstraat 14, 1050 Brussels, Belgium; 2 Centre for Environmental Sciences, Hasselt University, 3500 Hasselt, Belgium; 3 Sciensano, Epidemiology and Public Health, Juliette Wytsmanstraat 14, 1050 Brussels, Belgium; 4 Centre for Environment and Health, Leuven University, 3000 Leuven, Belgium

**Keywords:** Mental health, Lifestyle, Biological ageing, Mitochondrial DNA content, Telomere length

## Abstract

**Background:**

Studies often evaluate mental health and well-being in association with individual health behaviours although evaluating multiple health behaviours that co-occur in real life may reveal important insights into the overall association. Also, the underlying pathways of how lifestyle might affect our health are still under debate. Here, we studied the mediation of different health behaviours or lifestyle factors on mental health and its effect on core markers of ageing: telomere length (TL) and mitochondrial DNA content (mtDNAc).

**Methods:**

In this study, 6054 adults from the 2018 Belgian Health Interview Survey (BHIS) were included. Mental health and well-being outcomes included psychological and severe psychological distress, vitality, life satisfaction, self-perceived health, depressive and generalised anxiety disorder and suicidal ideation. A lifestyle score integrating diet, physical activity, smoking status, alcohol consumption and BMI was created and validated. On a subset of 739 participants, leucocyte TL and mtDNAc were assessed using qPCR. Generalised linear mixed models were used while adjusting for a priori chosen covariates.

**Results:**

The average age (SD) of the study population was 49.9 (17.5) years, and 48.8% were men. A one-point increment in the lifestyle score was associated with lower odds (ranging from 0.56 to 0.74) for all studied mental health outcomes and with a 1.74% (95% CI: 0.11, 3.40%) longer TL and 4.07% (95% CI: 2.01, 6.17%) higher mtDNAc. Psychological distress and suicidal ideation were associated with a lower mtDNAc of − 4.62% (95% CI: − 8.85, − 0.20%) and − 7.83% (95% CI: − 14.77, − 0.34%), respectively. No associations were found between mental health and TL.

**Conclusions:**

In this large-scale study, we showed the positive association between a healthy lifestyle and both biological ageing and different dimensions of mental health and well-being. We also indicated that living a healthy lifestyle contributes to more favourable biological ageing.

**Supplementary Information:**

The online version contains supplementary material available at 10.1186/s12916-022-02524-9.

## Background

According to the World Health Organization (WHO), a healthy lifestyle is defined as “a way of living that lowers the risk of being seriously ill or dying early” [1]. Public health authorities emphasise the importance of a healthy lifestyle, but despite this, many individuals worldwide still live an unhealthy lifestyle [2]. In Europe, 26% of adults smoke [3], nearly half (46%) never exercise [4], 8.4% drink alcohol on a daily basis [5] and over half (51%) are overweight [5]. These unhealthy behaviours have been associated with adverse health outcomes like cardiovascular diseases [6–8], respiratory diseases [9], musculoskeletal diseases [10] and, to a lesser extent, mental disorders [11, 12].

Even though the association between lifestyle and health outcomes has been extensively investigated, biological mechanisms explaining these observed associations are not yet fully understood. One potential mechanism that can be suggested is biological ageing. Both telomere length (TL) and mitochondrial DNA content (mtDNAc) are known biomarkers of ageing. Telomeres are the end caps of chromosomes and consist of multiple TTAGGG sequence repeats. They protect chromosomes from degradation and shorten with every cell division because of the “end-replication problem” [13]. Mitochondria are crucial to the cell as they are responsible for apoptosis, the control of cytosolic calcium levels and cell signalling [14]. Living a healthy lifestyle can be linked with healthy ageing as both TL and mtDNAc have been associated with health behaviours like obesity [15], diet [16], smoking [17] and alcohol abuse [18]. Furthermore, as biomarkers of ageing, both TL and mtDNAc have been associated with age-related diseases like Parkinson’s disease [19], coronary heart disease [20], atherosclerosis [21] and early mortality [22]. Also, early mortality and higher risks for the aforementioned age-related diseases are observed in psychiatric illnesses, and it is suggested that advanced biological ageing underlies these observations [23].

Multiple studies evaluated individual health behaviours, but research on the combination of these health behaviours is limited. As they often co-occur and may cause synergistic effects, assessing them in combination with each other rather than independently might better reflect the real-life situation [24, 25]. Therefore, in a general adult population, we combined five commonly studied health behaviours including diet, smoking status, alcohol consumption, BMI and physical activity into one healthy lifestyle score to evaluate its association with mental health and well-being and biological ageing. Furthermore, we evaluated the association between the markers of biological ageing and mental health and well-being. We hypothesise that individuals living a healthy lifestyle have a better mental health status, a longer TL and a higher mtDNAc and that these biomarkers are positively associated with mental health and well-being.

## Methods

### Study population

In 2018, 11611 Belgian residents participated in the 2018 Belgian Health Interview Survey (BHIS). The sampling frame of the BHIS was the Belgian National Register, and participants were selected based on a multistage stratified sampling design including a geographical stratification and a selection of municipalities within provinces, of households within municipalities and of respondents within households [26]. The study population for this cross-sectional study included 6054 BHIS participants (see flowchart in Additional file 1: Fig. S1) [27–31]. Minors (< 18 years) and participants not eligible to complete the mental health modules (participants who participated through a proxy respondent, i.e. a person of confidence filled out the survey) were excluded (*n* = 2172 and *n* = 846, respectively). Furthermore, of the 8593 eligible participants, those with missing information to create the mental health indicators, the lifestyle score or the covariates used in this study were excluded (*n* = 1642, 788 and 109, respectively).

For the first time in 2018, a subset of 1184 BHIS participants contributed to the 2018 Belgian Health Examination Survey (BELHES). All BHIS participants were invited to participate except for minors (< 18 years), BHIS participants who participated through a proxy respondent and residents of the German Community of Belgium, the latter representing 1% of the Belgian population. Participants were recruited on a voluntary basis until the regional quotas were reached (450, 300 and 350 in respectively Flanders, Brussels Capital Region and Wallonia). These participants underwent a health examination, including anthropological measurements and completed an additional questionnaire. Also, blood and urine samples were collected. Of the 6054 included BHIS participants, 909 participated in the BELHES. Participants for whom we could not calculate both TL and mtDNAc were excluded (*n* = 170). More specifically, participants were excluded because they did not provide a blood sample (*n* = 91) or because they did not provide permission for DNA research (*n* = 32). Twenty samples were excluded from DNA extraction because either total blood volume was too low (*n* = 7), samples were clothed (*n* = 1) or tubes were broken due to freezing conditions (*n* = 12). Twenty-seven samples were excluded because they did not meet the biomarker quality control criteria (high technical variation in qPCR triplicates). This was not met for 3 TL samples, 20 mtDNAc samples and 4 samples for both biomarkers. For this subset, we ended up with a final number of 739 participants. Further in this paper, we refer to “the BHIS subset” for the BHIS participants (*n* = 6054) and the “BELHES subset” for the BELHES participants (*n* = 739).

As part of the BELHES, this project was approved by the Medical Ethics Committee of the University Hospital Ghent (registration number B670201834895). The project was carried out in line with the recommendations of the Belgian Privacy Commission. All participants have signed a consent form that was approved by the Medical Ethics Committee.

### Health interview survey

The BHIS is a comprehensive survey which aims to gain insight into the health status of the Belgian population. The questions on the different dimensions of mental health and well-being were based on international standardised and validated questionnaires [32], and this resulted in eight mental health outcomes that were used in this study. Detailed information on each indicator score and its use is addressed in Additional file 1: Table. S1. Firstly, the General Health Questionnaire (GHQ-12) provides the prevalence of psychological and severe psychological distress in the population [27]. On the total GHQ score, cut-off points of + 2 and + 4 were used to identify respectively psychological and severe psychological distress.

Secondly, we used two indicators for the positive dimensions of mental health: vitality and life satisfaction. Four questions of the short form health survey (SF-36) indicate the participant’s vital energy level [28, 33]. We used a cut-off point to identify participants with an optimal vitality score, which is a score equal to or above one standard deviation above the mean, as used in previous studies [34, 35]. Life satisfaction was measured by the Cantril Scale, which ranges from 0 to 10 [29]. A cut-off point of + 6 was used to indicate participants with high or medium life satisfaction versus low life satisfaction.

Thirdly, the question “How is your health in general? Is it very good, good, fair, bad or very bad?” was used to assess self-perceived health, also known as self-rated health. Based on WHO recommendations [36], the answer categories were dichotomised into “good to very good self-perceived health” and “very bad to fair self-perceived health”.

Fourthly, depressive and generalised anxiety disorders were defined using respectively the Patient Health Questionnaire (PHQ-9) and the Generalised Anxiety Disorder Questionnaire (GAD-7). We identified individuals who suffer from major depressive syndrome or any other type of depressive syndrome according to the criteria of the PHQ-9 [37]. A cut-off point of + 10 on the total sum of the GAD-7 score was used to indicate generalised anxiety disorder [31]. Additionally, a dichotomous question on suicidal ideation was used: “Have you ever seriously thought of ending your life?”; “If yes, did you have such thoughts in the past 12 months?”. Finally, the BHIS also includes personal, socio-economic and lifestyle information. The standardised Cronbach’s alpha coefficients for the PHQ-9, GHQ-12, GAD-7 and questions on vitality of the SF-36 ranged between 0.80 and 0.90.

### Healthy lifestyle score

We developed a healthy lifestyle score based on five different health behaviours: body mass index (BMI), smoking status, physical activity, alcohol consumption and diet (Table 1). These health behaviours were defined as much as possible according to the existing guidelines for healthy living issued by the Belgian Superior Health Council [38] and the World Health Organisation [39–41]. Firstly, BMI was calculated as a person’s self-reported weight in kilogrammes divided by the square of the person’s self-reported height in metres (kg/m^2^). BMI was classified into four categories: underweight (BMI < 18.5 kg/m^2^), normal weight (BMI 18.5–24.9 kg/m^2^), overweight (BMI 25.0–29.9 kg/m^2^) and obese (BMI ≥ 30.0 kg/m^2^). Due to a J-shaped association of BMI with the overall mortality and multiple specific causes of death, obesity and underweight were both classified as least healthy [42]. BMI was scored as follows: obese and underweight = 0, overweight = 1 and normal weight = 2.

**Table 1:**
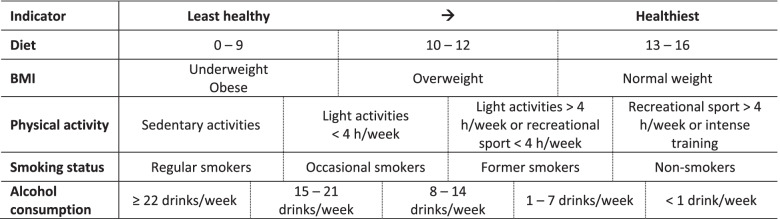
Healthy lifestyle score, where each health behaviour is scored from the least healthy to the healthiest

Secondly, smoking status was divided into four categories. Participants were categorised as regular smokers if they smoked a minimum of 4 days per week or if they quit smoking less than 1 month before participation (= 0). Occasional smokers were defined as smoking more than once per month up to 3 days per week (= 1). Participants were classified as former smokers if they quit smoking at least 1 month before the questionnaire or if they smoked less than once a month (= 2). The final category included people who never smoked (= 3).

Thirdly, physical activity was assessed by the question: “What describes best your leisure time activities during the last year?”. Four categories were established and scored as follows: sedentary activities (= 0), light activities less than 4 h/week (= 1), light activities more than 4 h/week or recreational sport less than 4 h/week (= 2) and recreational sport more than 4 h or intense training (= 3). Fourthly, information on the number of alcoholic drinks per week was used to categorise alcohol consumption. The different categories were set from high to low alcohol consumption: 22 drinks or more/week (= 0), 15–21 drinks/week (= 1), 8–14 drinks/week (= 2), 1–7 drinks/week (= 3)and less than 1 drink/week (= 4).

Finally, in line with the research by Benetou et al., a diet score was calculated using the frequency of consuming fruit, vegetables, snacks and sodas [43]. For fruit as well as vegetable consumption, the frequency was scored as follows: never (= 0), < 1/week (= 1), 1–3/week (= 2), 4–6/week (= 3) and ≥ 1/day (= 4). The frequency of consuming snacks and sodas was scored as follows: never (= 4), < 1/week (= 3), 1–3/week (= 2), 4–6/week (= 1) and ≥ 1/day (= 0). The diet score was then divided into tertiles, in line with the research by Benetou et al. [43]. A diet score of 0–9 points was classified as the least healthy behaviour (= 0). A diet score ranging from 10 to 12 made up the middle category (= 1), and a score from 13 to 16 was classified as the healthiest behaviour (= 2).

All five previously described health behaviours were combined into one healthy lifestyle score (Table 1). The sum of the scores obtained for each health behaviour indicated the absolute lifestyle score. To calculate the relative lifestyle score, each absolute scored health behaviour was given equal weight by recalculating its maximum absolute score to a relative score of 1. The relative lifestyle scores were then summed up to achieve a final continuous lifestyle score, ranging from 0 to 5, with a higher score representing a healthier lifestyle.

### Telomere length and mitochondrial DNA content assay

Blood samples were collected during the BELHES and centrifuged for 15 min at 3000 rpm before storage at − 80 °C. After extracting the buffy coat from the blood sample, DNA was isolated using the QIAgen Mini Kit (Qiagen, N.V.V Venlo, The Netherlands). The purity and quantity of the sample were measured with a NanoDrop spectrophotometer (ND-2000; Thermo Fisher Scientific, Wilmington, DE, USA). DNA integrity was assessed by agarose gel electrophoresis. To ensure a uniform DNA input of 6 ng for each qPCR reaction, samples were diluted and checked using the Quant-iT™ PicoGreen® dsDNA Assay Kit (Life Technologies, Europe).

Relative TL and mtDNAc were measured in triplicate using a previously described quantitative real-time PCR (qPCR) assay with minor modifications [44, 45]. All reactions were performed on a 7900HT Fast Real-Time PCR System (Applied Biosystems, Foster City, CA, USA) in a 384-well format. Used telomere, mtDNAc and single copy-gene reaction mixtures and PCR cycles are given in Additional file 1: Text. S1. Reaction efficiency was assessed on each plate by using a 6-point serial dilution of pooled DNA. Efficiencies ranged from 90 to 100% for single-copy gene runs, 100 to 110% for telomere runs and 95 to 105% for mitochondrial DNA runs. Six inter-run calibrators (IRCs) were used to account for inter-run variability. Also, non-template controls were used in each run. Raw data were processed and normalised to the reference gene using the qBase plus software (Biogazelle, Zwijnaarde, Belgium), taking into account the run-to-run differences.

Leucocyte telomere length was expressed as the ratio of telomere copy number to single-copy gene number (T/S) relative to the mean T/S ratio of the entire study population. Leucocyte mtDNAc was expressed as the ratio of mtDNA copy number to single-copy gene number (M/S) relative to the mean M/S ratio of the entire study population. The reliability of our assay was assessed by calculating the interclass correlation coefficient (ICC) of the triplicate measures (T/S and M/S ratios and T, M and S separately) as proposed by the Telomere Research Network, using RStudio version 1.1.463 (RStudio PBC, Boston, MA, USA). The intra-plate ICCs of T/S ratios, TL runs, M/S ratios, mtDNAc runs and single-copy runs were respectively 0.804 (*p* < 0.0001), 0.907 (*p* < 0.0001), 0.815 (*p* < 0.0001), 0.916 (*p* < 0.0001) and 0.781 (*p* < 0.0001). Based on the IRCs, the inter-plate ICC was 0.714 (*p* < 0.0001) for TL and 0.762 (*p* < 0.0001) for mtDNAc.

### Statistical analysis

Statistical analyses were performed using the SAS software (version 9.4; SAS Institute Inc., Cary, NC, USA). We performed a log(10) transformation of the TL and mtDNAc data to reduce skewness and to better approximate a normal distribution. Three analyses were done: (1) In the BHIS subset (*n* = 6054), we evaluated the association between the lifestyle score and the mental health and well-being outcomes (separately). These results are presented as the odds ratio (95% CI) of having a mental health condition or disorder for a one-point increment in the lifestyle score. (2) In the BELHES subset (*n* = 739), we evaluated the association between the lifestyle score and both TL and mtDNAc (separately). These results are presented as the percentage difference in TL or mtDNAc (95% CI) for a one-point increment in the lifestyle score. (3) In the BELHES subset (*n* = 739), we evaluated the association between the mental health and well-being outcomes and both TL and mtDNAc (separately). These results are presented as the percentage difference in TL or mtDNAc (95% CI) when having a mental health condition or disorder compared with the healthy group.

For all three analyses, we performed multivariable linear mixed models (GLIMMIX; unstructured covariance matrix) taking into account a priori selected covariates including age (continuous), sex (male, female), region (Flanders, Brussels Capital Region, Wallonia), highest educational level of the household (up to lower secondary, higher secondary, college or university), country of birth (Belgium, EU, non-EU) and household type (single, one parent with child, couple without child, couple with child, others). To capture the non-linear effect of age, we included a quadratic term when the result of the analysis showed that both the linear and quadratic terms had a *p*-value < 0.1. For the two analyses on TL and mtDNAc, we additionally adjusted for the date of participation in the BELHES. As multiple members of one household participated, we added household numbers in the random statement.

Bivariate analyses evaluating the associations between the characteristics and TL, mtDNAc, the lifestyle score or psychological distress as a parameter of mental health and well-being are evaluated based on the same model. The chi-squared tests (categorical data) and *t*-tests (continuous data) were used to evaluate the characteristics of included and excluded participants. The lifestyle score was validated by creating a ROC curve and calculating the area under the curve (AUC) of the adjusted association between the lifestyle score and self-perceived health. Adjustments were made for age, sex, region, highest educational level of the household, country of birth and household type.

In a sensitivity analysis, to evaluate the robustness of our findings, we additionally adjusted our main models separately for perceived quality of social support (poor, moderate, strong) and chronic disease (suffering from any chronic disease or condition: yes, no). The third model, evaluating the biomarkers with the mental health outcomes, was also additionally adjusted for the lifestyle score.

## Results

### Population characteristics

The characteristics of the BHIS and BELHES subset are presented in Table 2. In the BHIS subset, 48.8% of the participants were men. The average age (SD) was 49.9 (17.5) years, and most participants were born in Belgium (79.5%). The highest educational level in the household was most often college or university degree (53.3%), and the most common household composition was couple with child(ren) (37.7%). The proportion of participants in different regions of Belgium, i.e. Flanders, Brussels Capital Region and Wallonia, was respectively 41.1%, 23.3% and 35.6%. For the BELHES subset, we found similar results except for region and education. We noticed more participants from Flanders and more participants with a high educational level in the household. The mean (SD) relative TL and mtDNAc were respectively 1.04 (0.23) and 1.03 (0.24). TL and mtDNAc were positively correlated (Spearman’s correlation = 0.21, *p* < 0.0001).

**Table 2:** Characteristics of the study population for the BHIS (*n* = 6054) and the BELHES subset (*n* = 739)

Characteristics	BHIS subset, *n* (%) or mean (SD)	BELHES subset, *n* (%) or mean (SD)
Male	2955 (48.8%)	369 (49.9%)
Age, years	49.9 (17.5)	48.3 (15.5)
Region		
Flanders	2488 (41.1%)	356 (48.2%)
Brussels Capital Region	1410 (23.3%)	158 (21.4%)
Wallonia	2156 (35.6%)	225 (30.5%)
Highest educational level in the household		
Up to lower secondary school	1010 (16.7%)	92 (12.5%)
Higher secondary school	1819 (30.1%)	196 (26.5%)
College or university	3225 (53.3%)	451 (61.0%)
Household composition		
Single	1339 (22.1%)	130 (17.6%)
One parent with a child	514 (8.5%)	53 (7.2%)
Couple without child	1674 (27.7%)	196 (26.5%)
Couple with child(ren)	2283 (37.7%)	326 (44.1%)
Others	244 (4.0%)	34 (4.6%)
Country of birth		
Belgium	4812 (79.5%)	596 (80.7%)
EU	619 (10.2%)	77 (10.4%)
Non-EU	623 (10.3%)	66 (8.9%)
Perceived quality of social support^a^		
Poor	946 (15.7%)	116 (15.9%)
Moderate	2978 (49.5%)	379 (51.9%)
Strong	2093 (34.8%)	236 (32.3%)
Chronic disease or condition^b^	1776 (29.5%)	206 (28.1%)

^a^*n* = 6017 and 731 for the BHIS and BELHES subset, respectively

^b^*n* = 6017 and 733 for the BHIS and BELHES subset, respectively

We compared (1) the characteristics of the 6054 eligible BHIS participants that were included in the BHIS subset with the 2539 eligible participants that were excluded from the BHIS subset (Additional file 1: Table S2) and (2) the 739 participants from the BHIS subset that were included in the BELHES subset with the 5315 participants that were excluded from the BELHES subset (Additional file 1: Table S3). Except for sex and nationality in the latter, all other covariates showed differences between the included and excluded groups. On the other hand, population data from 2018 indicates that the average age (SD) of the adult Belgian population was 49.5 (18.9) with a distribution over Flanders, Brussels Capital Region and Wallonia of respectively 58.2%, 10.2% and 31.6% and that 48.7% were men. The distribution of our sample according to age and sex thus largely corresponds to the age and sex distribution of the adult Belgian population figures. The large difference in the regional distribution is due to the oversampling of the Brussels Capital Region in the BHIS.

Bivariate associations evaluating the characteristics with TL, mtDNAc, the lifestyle score or psychological distress as a parameter of mental health are presented in Additional file 1: Table S4. Briefly, men had a − 6.41% (95% CI: − 9.10 to − 3.65%, *p* < 0.0001) shorter TL, a − 8.03% (95% CI: − 11.00 to − 4.96%, *p* < 0.0001) lower mtDNAc, lower odds of psychological distress (OR = 0.59, 95% CI: 0.53 to 0.66, *p* < 0.0001) and a lifestyle score of − 0.28 (95% CI: − 0.32 to − 0.24, *p* < 0.0001) points less compared with women. Furthermore, a 1-year increment in age was associated with a − 0.64% (− 0.73 to − 0.55%, *p* < 0.0001) shorter TL and a − 0.19% (95% CI: − 0.31 to − 0.08%, *p* = 0.00074) lower mtDNAc.

### Mental health prevalence and lifestyle characteristics

Within the BHIS subset, 32.3% and 18.0% of the participants had respectively psychological and severe psychological distress. 86.7% had suboptimal vitality, 12.0% indicated low life satisfaction and 22.0% had very bad to fair self-perceived health. The prevalence of depressive and generalised anxiety disorders was respectively 9.0% and 10.8%, respectively. 4.4% of the participants indicated to have had suicidal thoughts in the past 12 months. Similar results were found for the BELHES subset (Table 3).

**Table 3:** Prevalence of the mental health and well-being outcomes for the BHIS (*n* = 6054) and the BELHES subset (*n* = 739)

Mental health and well-being outcomes	BHIS subset, *n* (%)	BELHES subset, *n* (%)
Psychological distress	1954 (32.3%)	252 (34.1%)
Severe psychological distress	1087 (18.0%)	131 (17.7%)
Suboptimal vitality	5247 (86.7%)	661 (89.5%)
Low life satisfaction	726 (12.0%)	82 (11.1%)
Very bad to fair self-perceived health	1333 (22.0%)	135 (18.3%)
Depressive disorder	544 (9.0%)	63 (8.5%)
Generalised anxiety disorder	655 (10.8%)	76 (10.3%)
Suicidal ideation	269 (4.4%)	38 (5.1%)

Within the BHIS subset, the average lifestyle score (SD) was 3.1 (0.9) (Table 4). A histogram of the lifestyle score is shown in Additional file 1: Fig. S2. 16.6% were regular smokers, and 4.9% reported 22 alcoholic drinks per week or more. 29.7% reported that their main leisure time included mainly sedentary activities, and 18.6% were underweight or obese. 29.2% were classified as having an unhealthy diet score. The participants of the BELHES subset were slightly more active, but no other dissimilarities were found (Table 4). The ROC curve shows an area under the curve (AUC) of 0.74, indicating a 74% predictive accuracy for the lifestyle score as a self-perceived health predictor (Additional file 1: Fig. S3).

**Table 4:** Characteristics of the healthy lifestyle score for the BHIS subset (*n* = 6054) and the BELHES subset (*n* = 739)

	**BHIS subset, *n* (%) or mean (SD)**	**BELHES subset, *n* (%) or mean (SD)**
Lifestyle score	3.1 (0.9)	3.1 (0.9)
Smoking status		
Regular smoker (0)	1002 (16.6%)	129 (17.5%)
Occasional smoker (1)	159 (2.6%)	25 (3.4%)
Former smoker (2)	1472 (24.3%)	180 (24.4%)
Nonsmoker (3)	3421 (56.5%)	405 (54.8%)
BMI		
Underweight/obese (0)	1123 (18.6%)	119 (16.1%)
Overweight (1)	2073 (34.2%)	248 (33.6%)
Normal weight (2)	2858 (47.2%)	372 (50.3%)
Physical activity		
Sedentary activities (0)	1795 (29.7%)	185 (25.0%)
Light activities < 4 h/week (1)	1391 (23.0%)	167 (22.6%)
Light activities > 4 h/week or sport < 4 h/week (2)	1820 (30.1%)	227 (30.7%)
Sport > 4 h/week or intense training (3)	1048 (17.3%)	160 (21.7%)
Alcohol consumption		
≥ 22 drinks/week (0)	294 (4.9%)	31 (4.2%)
15–21 drinks/week (1)	323 (5.3%)	52 (7.0%)
8–14 drinks/week (2)	738 (12.2%)	101 (13.7%)
1–7 drinks/week (3)	1777 (29.4%)	235 (31.8%)
< 1 drink/week or abstainers (4)	2922 (48.3%)	320 (43.3%)
Diet		
Diet score 0–9 (0)	1769 (29.2%)	210 (28.4%)
Diet score 10–12 (1)	2621 (43.3%)	325 (44.0%)
Diet score 13–16 (2)	1664 (27.5%)	204 (27.6%)

### Healthy lifestyle and mental health and well-being

Living a healthier lifestyle, indicated by having a higher lifestyle score, was associated with lower odds of all mental health and well-being outcomes (Table 5). After adjustment, a one-point increment in the lifestyle score was associated with lower odds of psychological (OR = 0.74, 95% CI: 0.69, 0.79) and severe psychological distress (OR = 0.69, 95% CI: 0.64, 0.75). Similarly, for the same increment, the odds of suboptimal vitality, low life satisfaction and very bad to fair self-perceived health were respectively 0.62 (95% CI: 0.56, 0.68), 0.62 (95% CI: 0.56, 0.68) and 0.56 (95% CI: 0.52, 0.61). Finally, the odds of having depressive disorder, generalised anxiety disorder or suicidal ideation were respectively 0.57 (95% CI: 0.51, 0.63), 0.63 (95% CI: 0.57, 0.69) and 0.63 (95% CI: 0.55, 0.72) for a one-point increment in the lifestyle score.

**Table 5:** Associations between the lifestyle score and the mental health and well-being outcomes

Lifestyle score	OR	95% CI	*p*-value
Psychological distress	0.74	0.69, 0.79	< 0.0001
Severe psychological distress	0.69	0.64, 0.75	< 0.0001
Suboptimal vitality	0.62	0.56, 0.68	< 0.0001
Low life satisfaction	0.62	0.56, 0.68	< 0.0001
Very bad to fair self-perceived health	0.56	0.52, 0.61	< 0.0001
Depressive disorder	0.57	0.51, 0.63	< 0.0001
Generalised anxiety disorder	0.63	0.57, 0.69	< 0.0001
Suicidal ideation	0.63	0.55, 0.72	< 0.0001

Odds ratios (OR) and 95% confidence intervals (CI) for the different mental health and well-being outcomes for a one-point increment in the lifestyle score. Analyses were adjusted for age, sex, region, highest educational level in the household, household composition and country of birth

### The biomarkers of ageing

After adjustment, living a healthy lifestyle was positively associated with both TL and mtDNAc (Table 6). A one-point increment in the lifestyle score was associated with a 1.74 (95% CI: 0.11, 3.40%, *p* = 0.037) higher TL and a 4.07 (95% CI: 2.01, 6.17%, *p* = 0.00012) higher mtDNAc.

**Table 6:** Associations between the biomarkers and both the lifestyle score and the mental health and well-being outcomes

Lifestyle score/mental health and well-being outcome	TL			mtDNAc		
	**% difference**	**95% CI**	***p*-value**	**% difference**	**95% CI**	***p*-value**
Lifestyle score	1.74	0.11, 3.40	0.037	4.07	2.01, 6.17	0.00012
Psychological distress	− 0.12	− 3.04, 2.88	0.94	− 2.29	− 5.82, 1.36	0.21
Severe psychological distress	0.21	− 3.39, 3.94	0.91	− 4.62	− 8.85, − 0.20	0.041
Suboptimal vitality	− 3.26	− 7.46, 1.12	0.14	− 2.37	− 7.62, 3.17	0.39
Low life satisfaction	0.20	− 4.18, 4.79	0.93	− 2.00	− 7.30, 3.59	0.47
Very bad to fair self-perceived health	− 0.79	− 4.36, 2.90	0.67	− 2.53	− 6.87, 2.00	0.27
Depressive disorder	2.54	− 2.41, 7.73	0.32	2.84	− 3.28, 9.36	0.37
Generalised anxiety disorder	0.56	− 3.88, 5.20	0.81	2.05	− 3.52, 7.94	0.48
Suicidal ideation	0.73	− 5.44, 7.31	0.82	− 7.83	− 14.77, − 0.34	0.041

Difference (%) in relative telomere length (TL) and mitochondrial DNA content (mtDNAc) (with 95% CI) (1) for a one-point increment in the lifestyle score or (2) when having a mental health disorder or condition compared with the healthy group. Analyses were adjusted for age, sex, region, highest educational level in the household, household composition, country of birth and day of sample collection

People suffering from severe psychological distress had a − 4.62% (95% CI: − 8.85, − 0.20%, *p* = 0.041) lower mtDNAc compared with those who did not suffer from severe psychological distress. Similarly, people with suicidal ideation had a − 7.83% (95% CI: − 14.77, − 0.34%, *p* = 0.041) lower mtDNAc compared with those without suicidal ideation. No associations were found for the other mental health and well-being outcomes, and no associations were found between mental health and TL (Table 6).

### Sensitivity analysis

Additional adjustment of the main analyses for perceived quality of social support, chronic disease or lifestyle score (in the association between the mental health outcomes and the biomarkers of ageing) did not strongly change the effect of our observations (Additional file 1: Tables S5-S7). However, we noticed that most of the associations between severe psychological distress or suicidal ideation and mtDNAc showed marginally significant results.

## Discussion

In this study, we evaluated the associations between eight mental health and well-being outcomes, a healthy lifestyle score and 2 biomarkers of biological ageing: telomere length and mitochondrial DNA content. Having a healthy lifestyle was positively associated with all mental health and well-being indicators and the markers of biological ageing. Furthermore, having had suicidal ideation or suffering from severe psychological distress was associated with a lower mtDNAc. However, no association was found between mental health and TL.

### Healthy lifestyle and mental health and well-being

In the first part of this research, we evaluated the association between lifestyle and mental health and well-being and showed that living a healthy lifestyle was positively associated with better mental health and well-being outcomes. Similar trends were found in previous studies for each of the health behaviours separately [11, 12, 46–48]. Although evaluating these health behaviours separately provides valuable information, assessing them in combination with each other rather than independently might better reflect the real-life situation as they often co-occur and may exert a synergistic effect on each other [24, 25, 49]. For example, 68% of the adults in England engaged in two or more unhealthy behaviours [25]. Especially, smoking status and alcohol consumption co-occurred, but half of the studies in the review by Noble et al. indicated clustering of all included health behaviours [24].

To date, the number of studies evaluating the combination of multiple health behaviours and mental health and well-being in adults is limited, and most of them use a different methodology to assess this association [50–56]. Firstly, differences are found between the included health behaviours. Most studies included the four “SNAP” risk factors, i.e. smoking, poor nutrition, excess alcohol consumption and physical inactivity. Other health behaviours that were sometimes included were BMI/obesity, sleep duration/quality and psychological distress [50, 53, 54, 56]. Secondly, differences are found in the scoring of the health behaviours and the use of the lifestyle score. Whereas in this study the health behaviours were scored categorically, studies often dichotomised the health behaviours and/or the final lifestyle score [50, 52, 53, 56]. Also, two studies performed clustering [54, 55]. Health behaviours can cluster together at both ends of the risk spectrum, but less is known about the middle categories. This is avoided by using the cluster method where participants are clustered based on similar behaviours. On the other hand, a lifestyle score can be of better use and more easily be interpreted when aiming to compare healthy versus unhealthy lifestyles, as was the case for this study.

Despite these different methods, all previously mentioned studies show similar results. Together with our findings, which also support these results, this provides clear evidence that an unhealthy lifestyle is associated with poor mental health and well-being outcomes. Important to notice is that, like our research, most studies in this field have a cross-sectional design and are therefore not able to assume causality. Therefore, mental health might be the cause or the consequence of an unhealthy lifestyle. Further prospective and longitudinal studies are warranted to confirm the direction of the association.

### Healthy lifestyle and biomarkers of ageing

How lifestyle affects our health is not yet fully understood. One possible pathway is through oxidative stress and biological ageing. An unhealthy lifestyle has been associated with an increase in oxidative stress [57–59], and in turn, higher concentrations of oxidative stress are known to negatively affect TL and mtDNAc [60]. In this study, we showed that living a healthy lifestyle was associated with a longer TL and a higher mtDNAc. Our results showed a stronger association of lifestyle with mtDNAc compared with TL. TL is strongly determined by TL at birth [61]. On the other hand, mtDNAc might be more variable in shorter time periods. Although mtDNAc and TL were strongly correlated, this could explain why lifestyle is more strongly associated with mtDNAc. However, we can only speculate about this, and further research is necessary to confirm our results.

Similar as for the association with mental health, in previous studies, the biomarkers have been associated with health behaviours separately rather than combined [62–65]. To our knowledge, we are the first to evaluate the associations between a healthy lifestyle score and mtDNAc. Our results are in line with our expectations. As TL and mtDNAc are known to be correlated [60], we would expect similar trends for both biomarkers. In the case of TL, few studies included a combined lifestyle score in association with this biomarker. Consistent with our results, in a study population of 1661 men, the sum score of a healthier lifestyle was correlated with a longer TL [66]. Similar results were found by Sun et al. where a combination of healthy lifestyles in a female study population was associated with a longer TL compared with the least healthy group [67]. Also, improvement in lifestyle has been associated with TL maintenance in the elderly at risk for dementia [68], and a lifestyle intervention programme was positively associated with leucocyte telomere length in children and adolescents [69]. These results suggest that on a biological level, a healthy lifestyle is associated with healthy ageing. Within this context, a study on adults aged 60 and older showed that maintaining a normal weight, not smoking and performing regular physical activity were associated with slower development of disability and a reduction in mortality [70]. Similarly, midlife lifestyle factors like non-smoking, higher levels of physical activity, non-obesity and good social support have been associated with successful ageing, 22 years later [71].

### Mental health and well-being and biomarkers of ageing

Finally, we evaluated the association between the biomarkers of ageing and the mental health and well-being outcomes. The hypothesis that biological ageing is associated with mental health has been supported by observations showing that chronically stressed or psychiatrically ill persons have a higher risk for age-related diseases like dementia, diabetes and hypertension [23, 72, 73]. Important to notice is that, like our research, the majority of studies on this topic have a cross-sectional design and therefore are unable to identify causality. Therefore, it is currently unknown whether psychological diseases accelerate biological ageing or whether biological ageing precedes the onset of these diseases [74].

Our results showed a lower mtDNAc for individuals with suicidal ideation or severe psychological distress but not for any of the other mental health outcomes. Evidence on the association between mtDNAc and mental health is inconsistent. Women above 60 years old with depression had a significantly lower mtDNAc compared with the control group [75]. Furthermore, individuals with a low mtDNAc had poorer outcomes in terms of self-rated health [76]. In contrast, Otsuka et al. showed a higher peripheral blood mtDNAc in suicide completers [77], and studies on major depressive syndrome [78] and self-rated health [79] showed the same trend. Finally, Vyas et al. showed no significant association between mtDNAc and depression status in mid-life and older adults [80]. These differences might be due to the differences in study population and methods. For example, the two studies indicating lower mtDNAc in association with poor mental health both had an elderly study population, and one study population consisted of psychiatrically ill patients. Next to that, differences were found in the type of samples, mtDNAc assays and questionnaires or diagnostics. The inconsistency of these studies and our results calls for further research on this association and for standardisation of methods within studies to enable clear comparisons.

As for TL, we did not find an association with any of the mental health and well-being outcomes. Previous studies in adults showed a lower TL in association with current but not remitted anxiety disorder [81], depressive [82] and major depressive disorder [73, 83], childhood trauma [84] suicide [77, 85], depressive symptoms in younger adults [86] and acculturative stress and postpartum depression in Latinx women [87]. Also, in a meta-analysis, psychiatric disorders overall were associated with a shorter leucocyte TL [88]. However, other studies failed to demonstrate an association between TL and mental health outcomes like major depressive disorder [89], late-life depression [90] and anxiety disorder [91]. Again, this could be due to a different method to assess the mental health outcomes, a different study design, uncontrolled confounding factors and the type of telomere assay. For example, a meta-analysis showed stronger associations with depression when using southern blot or FISH assay compared with qPCR to measure telomere length [92].

### Strengths and limitations

An important strength of this study is the use of a validated lifestyle score that can easily be reproduced and used for other research on lifestyle. Secondly, we were able to use a large sample size for our analyses in the BHIS subset. Thirdly, by assessing multiple dimensions of mental health and well-being, we were able to give a comprehensive overview of the mental health status. To our knowledge, we are the first to evaluate the associations between a healthy lifestyle score and mtDNAc.

Our results should however be interpreted with consideration for some limitations. As mentioned before, the study has a cross-sectional design, and therefore, we cannot assume causality. Secondly, for the lifestyle score, we used self-reported data, which might not always represent the actual situation. For example, BMI values tend to be underestimated due to the overestimation of height and underestimation of weight [93], and also, smoking behaviour is often underestimated [94]. Also, equal weights were used for each of the health behaviours as no objective information was available on which weight should be given to a specific health behaviour. Thirdly, there is a distinct time lag between the completion of the BHIS questionnaire and the collection of the BELHES samples. The mean (SD) number of days is 52 (35). This is less than the period for suicidal ideation, assessed over the 12 previous months, but there might be a more limited overlap with the period for assessment of the other mental health variables, such as vitality and psychological distress, assessed over the last few weeks, and depressive and generalised anxiety disorders, assessed over the last 2 weeks. Fourthly, due to a non-response bias, the lowest socio-economic classes are less represented in our study population. This will not affect our dose–response associations but might affect the generalisability of our findings to the overall population. Finally, we do not have data on blood cell counts, which has been associated with mtDNAc [95].

## Conclusions

In this large-scale study, we showed that living a healthy lifestyle was positively associated with mental health and well-being and, on a biological level, with a higher TL and mtDNAc, indicating healthy ageing. Furthermore, individuals with suicidal ideation or suffering from severe psychological distress had a lower mtDNAc. Our findings suggest that implementing strategies to incorporate healthy lifestyle changes in the public’s daily life could be beneficial for public health, and might offset the negative impact of environmental stressors. However, further studies are necessary to confirm our results and especially prospective and longitudinal studies are essential to determine causality of the associations.

## Supplementary Information

**Additional file 1: Text S1.** TL, mtDNAc and single copy-gene reaction mixture and PCR cycling conditions. **Table S1.** The mental health indicators with their scores and uses. **Table S2.** Comparison of the characteristics of the 6,054 eligible BHIS participants that were included in the BHIS subset compared to the 1,838 eligible participants that were excluded from the BHIS subset. **Table S3.** Comparison of the characteristics of the 739 participants from the BHIS subset that were included in the BELHES subset compared to the 5,315 participants that were excluded from the BELHES subset. **Table S4.** Bivariate associations between the characteristics and telomere length (TL), mitochondrial DNA content (mtDNAc), the lifestyle score or psychological distress. **Table S5.** Results of the sensitivity analysis of the association between lifestyle and mental health. **Table S6.** Results of the sensitivity analysis of the association between lifestyle and the biomarkers of ageing. **Table S7.** Results of the sensitivity analysis of the association between mental health and the biomarkers of ageing. **Fig. S1.** Exclusion criteria. The BHIS subset consisted of 6,055 BHIS participants and the BELHES subset consisted of 739 BELHES participants. **Fig. S2.** Histogram of the lifestyle score. **Fig. S3.** Validation of the lifestyle score. ROC curve for the lifestyle score as a predictor for good to very good self-perceived health. The model was adjusted for age, sex, region, highest educational level in the household, household composition and country of birth.

## Data Availability

The dataset used for this study is available through a request to the Health Committee of the Data Protection Authority.

## Abbreviations

AUC: Area under the curve
BMI: Body mass index
CI: Confidence intervals
GAD-7: Generalised Anxiety Disorder Questionnaire
GHQ-12: General Health Questionnaire
IRC: Inter-run calibrator
mtDNAc: Mitochondrial DNA content
OR: Odds ratio
PHQ-9: Patient Health Questionnaire
ROC curve: Relative operating characteristic curve
SF-36: Short Form Health Survey
TL: Telomere length
