# Supplementary material for: A healthy lifestyle is positively associated with mental health and well-being and core markers in ageing
Source: BMC Med. 2022 Sep 29;20:328. doi: 10.1186/s12916-022-02524-9 (PMC9520873; doi:10.1186/s12916-022-02524-9)
Supplement: Supplementary file 1 — Additional file 1: Text S1. TL, mtDNAc and single copy-gene reaction mixture and PCR cycling conditions. Table S1. The mental health indicators with their scores and uses. Table S2. Comparison of the characteristics of the 6,054 eligible BHIS participants that were included in the BHIS subset compared to the 1,838 eligible participants that were excluded from the BHIS subset. Table S3. Comparison of the characteristics of the 739 participants from the BHIS subset that were included in the BELHES subset compared to the 5,315 participants that were excluded from the BELHES subset. Table S4. Bivariate associations between the characteristics and telomere length (TL), mitochondrial DNA content (mtDNAc), the lifestyle score or psychological distress. Table S5. Results of the sensitivity analysis of the association between lifestyle and mental health. Table S6. Results of the sensitivity analysis of the association between lifestyle and the biomarkers of ageing. Table S7. Results of the sensitivity analysis of the association between mental health and the biomarkers of ageing. Fig. S1. Exclusion criteria. The BHIS subset consisted of 6,055 BHIS participants and the BELHES subset consisted of 739 BELHES participants. Fig. S2. Histogram of the lifestyle score. Fig. S3. Validation of the lifestyle score. ROC curve for the lifestyle score as a predictor for good to very good self-perceived health. The model was adjusted for age, sex, region, highest educational level in the household, household composition and country of birth. [file 12916_2022_2524_MOESM1_ESM.docx]

**Additional file 1 to:**

**A healthy lifestyle is positively associated with mental health and core markers in ageing**

Pauline Hautekiet^1,2^, Nelly D. Saenen^1,2^, Dries S. Martens^2^, Margot Debay^2^, Johan Van der Heyden^3^, Tim S. Nawrot^2,4^, Eva M. De Clercq^1^

^1^ Risk and health impact assessment, Sciensano, Juliette Wytsmanstraat 14, BE-1050, Brussels, Belgium

^2^ Centre for Environmental Sciences, Hasselt University, BE-3500, Hasselt, Belgium

^3^ Epidemiology and public health, Sciensano, Juliette Wytsmanstraat 14, BE-1050, Brussels, Belgium

^4^ Centre for Environment and Health, Leuven University, BE-3000, Leuven, Belgium

Corresponding author: Pauline Hautekiet, Juliette Wytsmanstraat 14, 1050 Brussels, Belgium, pauline.hautekiet@sciensano.be

**Text S1: TL, mtDNAc and single copy-gene reaction mixture and PCR cycling conditions.**

The TL reaction mixture contained Qiagen 2x QuantiTect SYBR Green Mastermix, 2 mM of dithiothreitol, 300 nM telg primer (ACACTAAGGTTTGGGTTTGGGTTTGGGTTTGGGTTAGTGT), 900 nM telc primer (TGTTAGGTATCCCTATCCCTATCCCTATCCCTATCCCTAACA) and 6 ng DNA. Thermal cycling profile was: 1 cycle of 10 min at 95°C, followed by 2 cycles of 15 s at 94°C and 2 min at 49°C, and finally 30 cycles of 15 s at 94°C, 20 s at 62°C and 1 min 40 s at 74°C.

The mtDNAc reaction mixture contained Qiagen 1x QuantiTect SYBR Green Mastermix, 300 nM ND1 forward primer (ATGGCCAACCTCCTACTCCT), 300 nM ND1 reverse primer (CTACAACGTTGGGGCCTTT) and 6 ng DNA. Thermal cycling profile was: 1 cycle of 10 min at 95°C, followed by 40 cycles of 15 s at 95°C and 1 min 10 s at 58°C.

The reference gene reaction mixture contained Qiagen 1x QuantiTect SYBR Green Mastermix, 400 nM HBG1 primer (GCTTCTGACACAACTGTGTTCACTAGC), 400 nM HBG2 (CACCAACTTCATCCACGTTCACC) primer and 6 ng DNA. Thermal cycling profile was: 1 cycle of 10 min at 95°C, followed by 40 cycles of 15 s at 95°C and 1 min 10 s at 58°C.

**Table S1: The mental health indicators with their scores and uses.**

| Questionnaire | Indicator | Score | Use |
| --- | --- | --- | --- |
| GHQ-12 | Indicator of mental well-being | 12 questions  Answers: ‘better than usual’, ‘as good as usual’, ‘less than usual’, ‘much less than usual’  Ranking: [0 0 1 1]  The sum (from 0-12) represents the global GHQ-score. | 1. A cut-off point of [2+] is used to identify participants with at least 2 ‘abnormal’ psychological complaints, indicating a possible psychopathology.  2. A cut-off point of [4+] is used to identify participants with at least 4 ‘abnormal’ psychological complaints, indicating the more severe cases. |
| SF-36 | Indicator of the positive psychological health (vitality) | 4 questions  Answers: ‘always’, ‘most of the time’, ‘sometimes’, ‘rarely’, ‘never’  Ranking: [5 4 3 2 1] for the first two questions and [1 2 3 4 5] for the last two questions.  The average of the scores is converted to a scale from 0 to 100. | The average score + the standard deviation is used as the cut-off point to indicate who has an vitality score well above average. |
| Cantril scale | Indicator of life satisfaction | On a scale from 0 to 10, where 0 means “not at all satisfied” and 10 means “completely satisfied”, how satisfied do you currently feel with your life as a whole? | A cut-off point of [+6] was used to indicate participants with a high or medium life satisfaction versus low life satisfaction. |
| PHQ-9 | Indicator of any type of depressive disorder | 9 questions  Answers: ‘not at all’, ‘several days’, ‘more than half the days’, ‘nearly every day’. | Depressive disorder is present when at least two questions are answered ‘more than half of the days’ and this at least for one of the first two questions. The last question (suicidal thoughts) is taken into account when answered ‘several days’. |
| GAD-7 | Indicator of general anxiety disorder | 7 questions  Answers: ‘not at all’, ‘several days’, ‘more than half the days’, ‘nearly every day’  Ranking [0 1 2 3]  The sum (from 0 to 21) represents the total score. | A cut-off point of [10+] is used to identify participants with a generalised anxiety disorder. |

**GHQ-12, General health questionnaire**

**SF-36, Short form health survey**

**PHQ-9, Patient Health Questionnaire 9-item depression scale**

**GAD-7, Generalised Anxiety Disorder questionnaire**

**Table S2: Comparison of the characteristics of the 6,054 eligible BHIS participants that were included in the BHIS subset compared to the 2,539 eligible participants that were excluded from the BHIS subset.**

| Characteristics | Included  n (%) or mean (SD) | Excluded  n (%) or mean (SD) | *p*-value |
| --- | --- | --- | --- |
| Male | 2,955 (48.8%) | 1,120 (44.1%) | < 0.0001 |
| Age, years | 49.9 (17.5) | 55.5 (18.3) | < 0.0001 |
| Region |  |  | < 0.0001 |
| Flanders | 2,488 (41.1%) | 759 (29.9%) |  |
| Brussels | 1,410 (23.3%) | 834 (32.9%) |  |
| Wallonia | 2,156 (35.6%) | 946 (37.3%) |  |
| Highest educational level in the household ^a^ |  |  | < 0.0001 |
| Up to lower secondary school | 1,010 (16.7%) | 726 (30.6%) |  |
| Higher secondary school | 1,819 (30.1%) | 757 (31.9%) |  |
| College or university | 3,225 (53.3%) | 890 (37.5%) |  |
| Household composition |  |  | < 0.0001 |
| Single | 1,339 (22.1%) | 741 (29.2%) |  |
| One parent with child | 514 (8.5%) | 217 (8.6%) |  |
| Couple without child | 1,674 (27.7%) | 745 (29.3%) |  |
| Couple with child(ren) | 2,283 (37.7%) | 695 (27.4%) |  |
| Other | 244 (4.0%) | 141 (5.6%) |  |
| Nationality ^b^ |  |  | < 0.0001 |
| Belgium | 4,812 (79.5%) | 1,711 (67.6%) |  |
| EU | 619 (10.2%) | 335 (13.2%) |  |
| Non-EU | 623 (10.3%) | 486 (19.2%) |  |

*p*-values showing the difference between the included and excluded groups are calculated with Chi-squared test (categorical data) and t-test (numerical data). ^a^ excluded: n = 2,373 and ^b^ excluded: n = 2,532

**Table S3: Comparison of the characteristics of the 739 participants from the BHIS subset that were included in the BELHES subset compared to the 5,315 participants that were excluded from the BELHES subset**.

| Characteristics | Included (n = 739)  n (%) or mean (SD) | Excluded (n = 5,315)  n (%) or mean (SD) | *p*-value |
| --- | --- | --- | --- |
| Male | 369 (49.9%) | 2586 (48.7%) | 0.52 |
| Age, years | 48.3 (15.5) | 50.2 (17.8) | 0.0021 |
| Region |  |  | 0.00013 |
| Flanders | 356 (48.2%) | 2,132 (40.1%) |  |
| Brussels Capital Region | 158 (21.4%) | 1,252 (23.6%) |  |
| Wallonia | 225 (30.5%) | 1,931 (36.3%) |  |
| Highest educational level in the household |  |  | < 0.0001 |
| Up to lower secondary school | 92 (12.5%) | 918 (17.3%) |  |
| Higher secondary school | 196 (26.5%) | 1,623 (30.5%) |  |
| College or university | 451 (61.0%) | 2,774 (52.2%) |  |
| Household composition |  |  | 0.00056 |
| Single | 130 (17.6%) | 1,209 (22.8%) |  |
| One parent with child | 53 (7.2%) | 461 (8.7%) |  |
| Couple without child | 196 (26.5%) | 1,478 (27.8%) |  |
| Couple with child(ren) | 326 (44.1%) | 1,957 (36.8%) |  |
| Other | 34 (4.6%) | 210 (4.0%) |  |
| Country of birth |  |  | 0.43 |
| Belgium | 596 (80.7%) | 4,216 (79.3%) |  |
| EU | 77 (10.4%) | 542 (10.2%) |  |
| Non-EU | 66 (8.9%) | 557 (10.5%) |  |

*p*-values showing the difference between the included and excluded groups are calculated with Chi-squared test (categorical data) and t-tests (numerical data).

+

**Table S4: Bivariate associations between the characteristics and telomere length (TL), mitochondrial DNA content (mtDNAc), the lifestyle score or psychological distress.**

| Characteristics | TL  % difference (95% CI) | *p*-value | mtDNAc  % difference (95% CI) | *p*-value | Lifestyle score  Estimate (95% CI) | *p*-value | Psychological distress  OR (95% CI) | *p*-value |
| --- | --- | --- | --- | --- | --- | --- | --- | --- |
| Age | -0.64 (-0.73, -0.55) | < 0.0001 | -0.19 (-0.31, -0.08) | 0.00074 | -0.0024 (-0.0037, -0.0011) | 0.00041 | 1.003 (1.002, 1.003) | < 0.0001 |
| Sex |  |  |  |  |  |  |  |  |
| Female | Ref |  | Ref |  | Ref |  | Ref |  |
| Male | -6.41 (-9.10, -3.65) | < 0.0001 | -8.03 (-11.00, -4.96) | < 0.0001 | -0.28 (-0.32, -0.24) | < 0.0001 | 0.59 (0.53, 0.66) | < 0.0001 |
| Highest educational level in the household |  |  |  |  |  |  |  |  |
| Up to lower secondary | -11.59 (-15.87, -7.10) | < 0.0001 | -7.31 (-12.19, -2.16) | 0.0062 | -0.41 (-0.48, -0.35) | < 0.0001 | 1.15 (0.98, 1.35) | 0.079 |
| Higher secondary school | -4.37 (-7.89, -0.71) | 0.020 | -0.95 (-4.90, 3.16) | 0.64 | -0.35 (-0.40, -0.29) | < 0.0001 | 0.98 (0.86, 1.12) | 0.78 |
| College or university | Ref |  | Ref |  | Ref |  | Ref |  |
| Country of birth |  |  |  |  |  |  |  |  |
| Belgium | Ref |  | Ref |  | Ref |  | Ref |  |
| EU | 3.40 (-1.90, 8.98) | 0.21 | 3.68 (-2.09, 9.78) | 0.21 | 0.12 (0.04, 0.20) | 0.0023 | 0.91 (0.75, 1.10) | 0.33 |
| Non-EU | 0.67 (-4.85, 6.51) | 0.81 | -0.59 (-6.50, 5.69) | 0.85 | 0.18 (0.10, 0.25) | < 0.0001 | 1.20 (1.00, 1.44) | 0.054 |
| Household type |  |  |  |  |  |  |  |  |
| Single | -9.03 (-12.97, -4.90) | < 0.0001 | -5.51 (-10.01, -0.78) | 0.023 | -0.19 (-0.25, -0.12) | < 0.0001 | 1.26 (1.09, 1.46) | 0.0024 |
| One parent with child | 1.95 (-4.31, 8.63) | 0.55 | 5.40 (-1.70, 13.02) | 0.14 | -0.08 (-0.18, 0.01) | 0.075 | 1.55 (1.26, 1.91) | < 0.0001 |
| Couple without child | -7.62 (-11.24, -3.85) | 0.00013 | -2.29 (-6.43, 2.04) | 0.29 | -0.07 (-0.14, -0.01) | 0.021 | 0.76 (0.65, 0.88) | 0.00024 |
| Couple with child(ren) | Ref |  | Ref |  | Ref |  | Ref |  |
| Other | 0.09 (-7.50, 8.31) | 0.98 | -0.56 (-8.75, 8.37) | 0.90 | -0.19 (-0.32, -0.05) | 0.0062 | 1.25 (0.93, 1.68) | 0.13 |
| Region |  |  |  |  |  |  |  |  |
| Flanders | Ref |  | Ref |  | Ref |  | Ref |  |
| Brussels capital region | -2.55 (-6.59, 1.68) | 0.23 | -3.09 (-7.41, 1.43) | 0.18 | -0.01 (-0.08, 0.05) | 0.65 | 1.43 (1.24, 1.66) | < 0.0001 |
| Wallonia | -4.36 (7.91, -0.67) | 0.021 | -0.51 (-4.47, 3.62) | 0.81 | -0.12 (-0.18, -0.06) | < 0.0001 | 1.25 (1.10, 1.43) | 0.00083 |

Mixed models were used adjusted for household number in the random statement. Results are presented as the % difference in TL or mtDNAc, a difference in lifestyle score or the odds of having psychological distress for a one-year increment in age or for a category compared to a reference category.

**Table S5: Results of the sensitivity analysis of the association between lifestyle and mental health.**

| Lifestyle score | + Perceived quality of social support  OR (95% CI) | *p*-value | + Chronic disease  OR (95% CI) | *p*-value |
| --- | --- | --- | --- | --- |
| Psychological distress | 0.78 (0.73, 0.84) | < 0.0001 | 0.78 (0.73, 0.84) | < 0.0001 |
| Severe psychological distress | 0.74 (0.68, 0.80) | < 0.0001 | 0.73 (0.67, 0.79) | < 0.0001 |
| Suboptimal vitality | 0.64 (0.58, 0.70) | < 0.0001 | 0.64 (0.58, 0.71) | < 0.0001 |
| Low life satisfaction | 0.67 (0.61, 0.74) | < 0.0001 | 0.65 (0.59, 0.72) | < 0.0001 |
| Very bad to fair self-perceived health | 0.59 (0.54, 0.64) | < 0.0001 | 0.58 (0.53, 0.63) | < 0.0001 |
| Depressive disorder | 0.61 (0.55, 0.68) | < 0.0001 | 0.60 (0.54, 0.67) | < 0.0001 |
| Generalized anxiety disorder | 0.67 (0.60, 0.74) | < 0.0001 | 0.66 (0.60, 0.73) | < 0.0001 |
| Suicidal ideation | 0.68 (0.59, 0.79) | < 0.0001 | 0.67 (0.58, 0.77) | < 0.0001 |

Odds ratios (OR) and 95% confidence intervals (CI) of the associations between the lifestyle score and the mental health outcomes additionally adjusted for perceived quality of social support or chronic disease (separately). Analyses were adjusted for age, sex, region, highest educational level in the household, household composition and country of birth.

**Table S6: Results of the sensitivity analysis of the association between lifestyle and the biomarkers of ageing.**

|  | TL  % difference (95% CI) | *p*-value | MtDNAc  % difference (95% CI) | *p*-value |
| --- | --- | --- | --- | --- |
| + Perceived quality of social support (n = 731) | 1.79 (0.14, 3.47) | 0.034 | 3.89 (1.82, 6.00) | 0.00025 |
| + Chronic disease (n = 733) | 1.74 (0.09, 3.42) | 0.039 | 4.07 (1.99, 6.18) | 0.00013 |

Difference (%) in telomere length (TL) and average relative mitochondrial DNA content (mtDNAc) (with 95% CI) for a one-point increment in lifestyle score additionally adjusted for perceived quality of social support or chronic disease (separately). Analyses were adjusted for age, sex, region, highest educational level in the household, household composition, country of birth and day of sample collection.

**Table S7: Results of the sensitivity analysis of the association between mental health and the biomarkers of ageing.**

|  | Mental health disorder | TL  % difference (95% CI) | *p*-value | MtDNAc  % difference (95% CI) | *p*-value |
| --- | --- | --- | --- | --- | --- |
| + Perceived quality of social support  (n = 731) | Psychological distress | -0.21 (-3.20, 2.86) | 0.89 | -1.89 (-5.51, 1.86) | 0.32 |
|  | Severe psychological distress | 0.24 (-3.47, 4.10) | 0.90 | -4.51 (-8.85, 0.04) | 0.052 |
|  | Suboptimal vitality | -3.21 (-7.44, 1.21) | 0.15 | -2.03 (-7.31, 3.55) | 0.47 |
|  | Low life satisfaction | 0.28 (-4.34, 5.12) | 0.91 | -1.32 (-6.91, 4.60) | 0.65 |
|  | Very bad to fair self-perceived health | -0.66 (-4.31, 3.13) | 0.73 | -2.14 (-6.56, 2.50) | 0.36 |
|  | Depressive disorder | 2.70 (-2.39, 8.05) | 0.30 | 3.75 (-2.57, 10.49) | 0.25 |
|  | Generalised anxiety disorder | 0.53 (-4.10, 5.39) | 0.82 | 2.53 (-3.29, 8.69) | 0.40 |
|  | Suicidal ideation | 0.29 (-6.04, 7.06) | 0.93 | -9.14 (-16.14, -1.55) | 0.020 |
| + Chronic disease | Psychological distress | -0.18 (-3.19, 2.92) | 0.91 | -2.14 (-5.78, 1.63) | 0.26 |
| (n = 733) | Severe psychological distress | 0.10 (-3.59, 3.93) | 0.96 | -4.46 (-8.79, 0.08) | 0.054 |
|  | Suboptimal vitality | -3.25 (-7.49, 1.19) | 0.15 | -2.09 (-7.39, 3.51) | 0.46 |
|  | Low life satisfaction | 0.11 (-4.36, 4.78) | 0.96 | -1.51 (-6.92, 4.22) | 0.60 |
|  | Very bad to fair self-perceived health | -1.30 (-5.16, 2.71) | 0.52 | -2.34 (-7.05, 2.61) | 0.35 |
|  | Depressive disorder | 2.90 (-2.19, 8.26) | 0.27 | 3.88 (-2.45, 10.62) | 0.23 |
|  | Generalised anxiety disorder | 0.79 (-3.80, 5.59) | 0.74 | 2.96 (-2.82, 9.09) | 0.32 |
|  | Suicidal ideation | 0.71 (-5.60, 7.46) | 0.83 | -7.01 (-14.15, 0.73) | 0.074 |
| + Lifestyle score | Psychological distress | 0.17 (-2.76, 3.19) | 0.91 | -1.53 (-5.07, 2.14) | 0.41 |
| (n = 739) | Severe psychological distress | 0.51 (-3.10, 4.26) | 0.78 | -3.89 (-8.12, 0.54) | 0.084 |
|  | Suboptimal vitality | -2.68 (-6.93, 1.77) | 0.23 | -0.83 (-6.17, 4.80) | 0.77 |
|  | Low life satisfaction | 0.30 (-4.08, 4.87) | 0.90 | -1.78 (-7.03, 3.77) | 0.52 |
|  | Very bad to fair self-perceived health | -0.11 (-3.75, 3.67) | 0.96 | -0.97 (-5.40, 3.67) | 0.68 |
|  | Depressive disorder | 2.93 (-2.03, 8.15) | 0.25 | 3.78 (-2.36, 10.30) | 0.23 |
|  | Generalised anxiety disorder | 0.67 (-3.76, 5.31) | 0.77 | 2.36 (-3.18, 8.23) | 0.41 |
|  | Suicidal ideation | 0.99 (-5.19, 7.57) | 0.76 | -7.24 (-14.15, 0.22) | 0.057 |

Difference (%) in telomere length (TL) and average relative mitochondrial DNA content (mtDNAc) (with 95% CI) when having a mental health disorder of condition compared to healthy group, adjusted for perceived quality of social support, hypertension or the lifestyle score (separately). Analyses were adjusted for age, sex, region, highest educational level in the household, household composition, country of birth and day of sample collection.

**
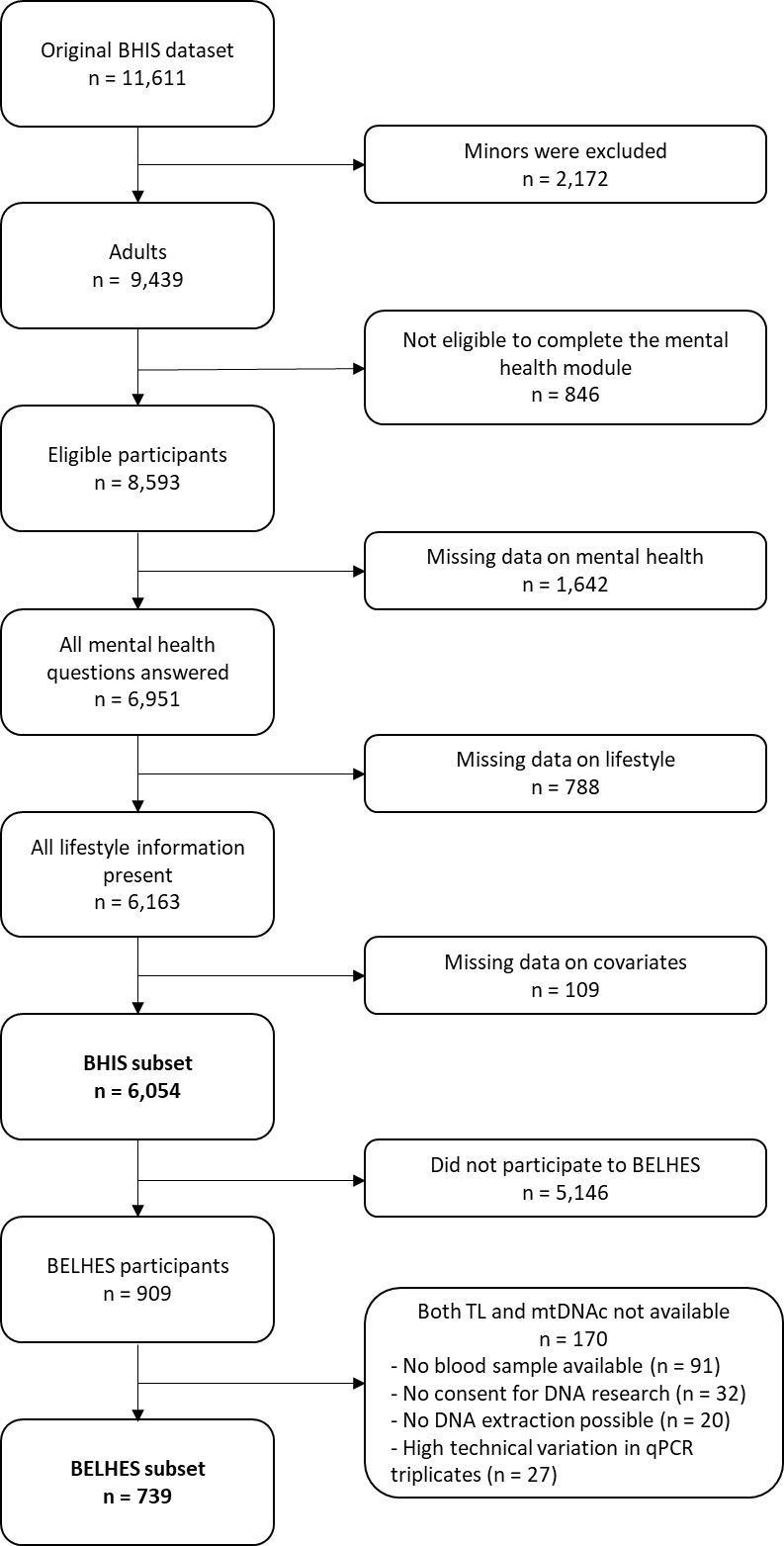
**

**Figure S1: Exclusion criteria.** The BHIS subset consisted of 6,054 BHIS participants and the BELHES subset consisted of 739 BELHES participants.


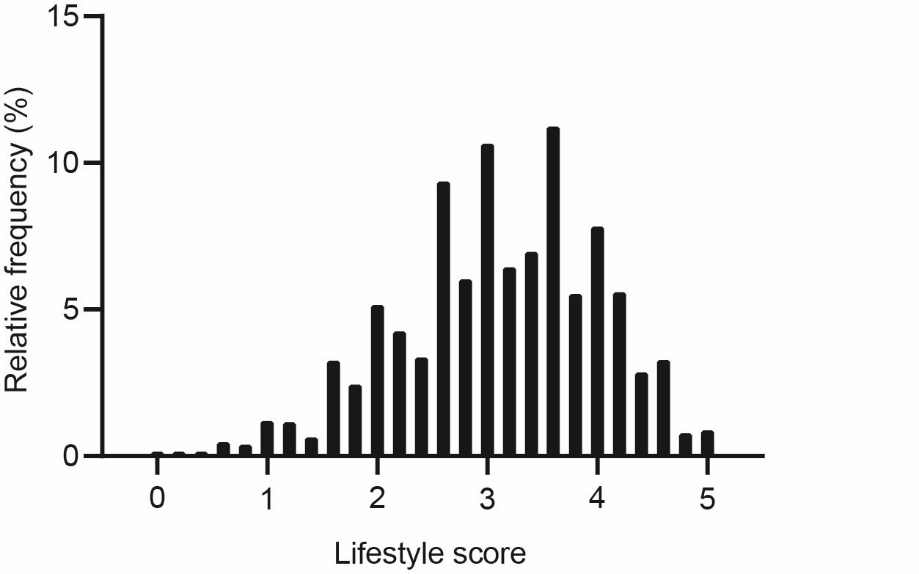


**Figure S2: Histogram of the lifestyle score.**


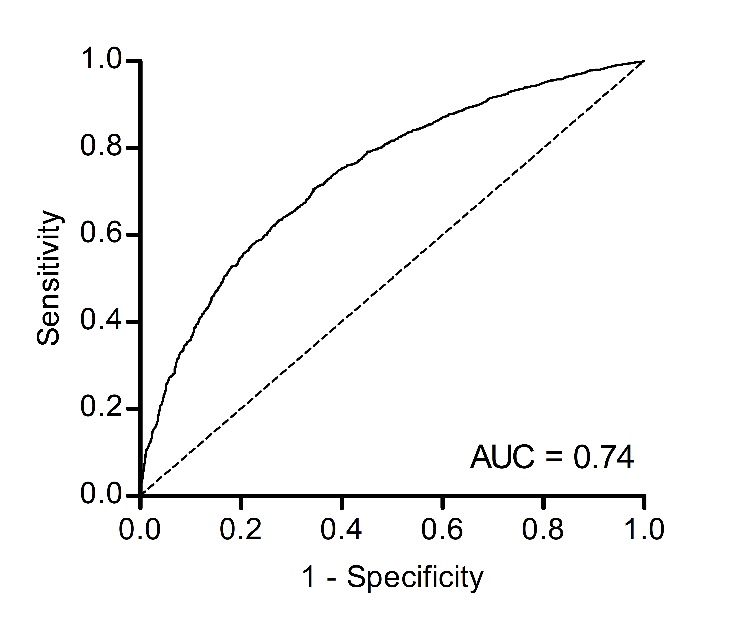


**Figure S3: Validation of the lifestyle score.** ROC curve for the lifestyle score as a predictor for good to very good self-perceived health. The model was adjusted for age, sex, region, highest educational level in the household, household composition and country of birth.
